# 356 Evaluation of Infectious Diseases Provider Ordering Practices Following Implementation of Prosthetic Joint Infection Guidelines

**DOI:** 10.1017/ash.2026.10694

**Published:** 2026-06-23

**Authors:** Jayme Bland, Nancy Kerr, Karen Pasco, Jill Armstrong, Cara Kellner, Laura Giambona, Sukrut Dwivedi

**Affiliations:** 1 Hackensack Meridian Ocean University Medical Center; 2 Ocean University Medical Center/Hackensack Meridian Health; 3 Ocean University Medical Center; 4 HMHN Ocean University Medical Center

## Abstract

**Background:** After an 18-month period with no Central Line-Associated Bloodstream Infections (CLABSIs), the Critical Care Unit (CCU) at OUMC experienced a cluster of two CLABSIs and one related non-CLABSI event in the first quarter of 2025. This occurred despite established prevention protocols, including daily line necessity rounds, use of MAGIC guidelines for vascular access, universal decolonization, and ongoing staff education, which had successfully lowered infection rates since 2022. All three affected patients were intubated, had an internal jugular (IJ) central venous catheter, and presented with Candida species in their blood and sputum, signaling a potential common etiology. **Methods:** In response, a multidisciplinary team was convened in April 2025 to conduct a proactive risk assessment using the Failure Modes and Effects Analysis (FMEA) methodology. The FMEA focused on ensuring adherence to the evidence-based policy for central line insertion and maintenance. The team comprised intensivists, interventional radiologists, chief residents, nursing leadership, infection prevention specialists, and a patient safety coordinator to represent all facets of the central line process. **Results:** The FMEA systematically evaluated potential failures in central line necessity, insertion, maintenance, removal, and environmental cleaning. By calculating Risk Profile Numbers (RPNs), the team identified several high-risk process steps. The highest initial RPN (486) was attributed to the potential for insufficient ultrasound probe cleaning due to improper technique and reliance on visual inspection alone. Other significant failure modes included inconsistent terminal cleaning of patient rooms (RPN 315), potential contamination of IJ lines from patient secretions (RPN 216), lack of role clarity during the insertion procedure (RPN 192), and the use of a single needle for multiple insertion attempts (RPN 189). The cumulative initial RPN for all identified failure modes was 2798. **Conclusion:** The FMEA proved to be an effective tool for identifying systemic vulnerabilities that contributed to the CLABSI cluster. The analysis led to the implementation of targeted corrective actions, including re-education on and audited monitoring of ultrasound probe cleaning, the introduction of single-use gel packets and individual introducer needles, clarification of the safety observer's role during insertion, and enhanced protocols for protecting IJ dressings. Following these interventions, the total RPN was successfully reduced to 1763. This proactive, systematic approach enabled the team to address specific gaps in practice and reinforce safety protocols to prevent future CLABSI events. No further CLABSIs have been identified since these interventions.

